# The impact of biochar addition on morpho-physiological characteristics, yield and water use efficiency of tomato plants under drought and salinity stress

**DOI:** 10.1186/s12870-024-05058-9

**Published:** 2024-05-09

**Authors:** Ghulam Murtaza, Muhammad Usman, Javed Iqbal, Muhammad Nauman Tahir, Mohamed S. Elshikh, Jawaher Alkahtani, Monika Toleikienė, Rashid Iqbal, M. Irfan Akram, Nazim S. Gruda

**Affiliations:** 1 Faculty of Environmental Science and Engineering, Kunming University of Science and Technology, Kunming, 650500 China; 2 School of Agriculture and Biology, Shanghai Jiao Tong University, 800 Dongchuan Road, Minghang District, Shanghai, 200240 China; 3 Department of Botany, Bacha Khan University, Charsadda, Khyber Pakhtunkhwa 24420 Pakistan; 4 Department of Botany and Microbiology, College of Science, King Saud University, Riyadh, 11451 Saudi Arabia; 5 Institute of Agriculture, Lithuanian Research Centre for Agriculture and Forestry, Instituo Al. 1, Akademija, Kedainiai, LT- 58344 Lithuania; 6 Department of Agronomy, Faculty of Agriculture and Environment, The Islamia University of Bahawalpur, Bahawalpur, 63100 Pakistan; 7 Department of Entomology, Faculty of Agriculture and Environment, The Islamia University of Bahawalpur, Bahawalpur, 63000 Pakistan; 8 Institute of Plant Sciences and Resource Conservation, Division of Horticultural Sciences, University of Bonn, 53115 Bonn, Germany

**Keywords:** Abiotic stress, Plant growth, Produce quality, Salinity, Biochar, *Solanum lycopersicum*

## Abstract

The use of saline water under drought conditions is critical for sustainable agricultural development in arid regions. Biochar is used as a soil amendment to enhance soil properties such as water-holding capacity and the source of nutrition elements of plants. Thus, the research was carried out to assess the impact of biochar treatment on the morphological and physiological characteristics and production of *Solanum lycopersicum* in greenhouses exposed to drought and saline stresses. The study was structured as a three-factorial in split-split-plot design. There were 16 treatments across three variables: (i) water quality, with freshwater and saline water, with electrical conductivities of 0.9 and 2.4 dS m^− 1^, respectively; (ii) irrigation level, with 40%, 60%, 80%, and 100% of total evapotranspiration (ETC); (iii) and biochar application, with the addition of biochar at a 3% dosage by (w/w) (BC_3%_), and a control (BC_0%_). The findings demonstrated that salt and water deficiency hurt physiological, morphological, and yield characteristics. Conversely, the biochar addition enhanced all characteristics. Growth-related parameters, such as plant height, stem diameter, leaf area, and dry and wet weight, and leaf gas exchange attributes, such rate of transpiration and photosynthesis, conductivity, as well as leaf relative water content were decreased by drought and salt stresses, especially when the irrigation was 60% ETc or 40% ETc. The biochar addition resulted in a substantial enhancement in vegetative growth-related parameters, physiological characteristics, efficiency of water use, yield, as well as reduced proline levels. Tomato yield enhanced by 4%, 16%, 8%, and 3% when irrigation with freshwater at different levels of water deficit (100% ETc, 80% ETc, 60% ETc, and 40% ETc) than control (BC_0%_). Overall, the use of biochar (3%) combined with freshwater shows the potential to enhance morpho-physiological characteristics, support the development of tomato plants, and improve yield with higher WUE in semi-arid and arid areas.

## Introduction

Tomato (*Solanum lycopersicum*) is widely recognized as one of the most commonly consumed vegetables globally [1]. Ensuring a substantial crop yield of tomatoes is crucial to address the growing need for food in Pakistan [2]. Tomato is abundant in minerals and antioxidants, including vitamin C, lycopene, and phenols [3]. Drought and salinity are the principal abiotic stresses that significantly restrict crop growth and yield on a global scale [4]. Bahawalpur is renowned for its arid climate, making it one of the most parched regions in Pakistan. Approximately 70% of water reserves are utilized for agricultural purposes, alongside several other elements that impact agricultural practices [5]. Much of the soil in Bahawalpur consists of sandy-loam and sand-based soils, characterized by a limited ability to retain water, a rapid rate of water penetration and a low level of clay. Consequently, these soils require meticulous treatment.

Irrigated agriculture consumes over two-thirds of global freshwater usage, making it the primary consumer of this resource [6]. Meeting the need for nutritious food for a growing global population while optimizing water usage for crop irrigation poses a significant challenge in agriculture nowadays [7]. The modern approach to water conservation focuses on enhancing water use efficiency while maintaining productivity levels [8]. *Solanum lycopersicum* plants, when exposed to drought stress, decrease the leaf area and photosynthetic level. This final result in a reduction in the accumulation of biomass and yield [9]. Pappula-Reddy et al. [10] found that water stress can result in yield reductions ranging from 13 to 94%, contingent upon the duration and intensity of the drought stress. Alza et al. [11] observed a 16% decrease in *Solanum lycopersicum* yield when exposed to a water deficit of 75% ETc compared to full irrigation. Nevertheless, drought-induced stress commonly decreases crop productivity and enhances water use efficiency, as demonstrated by [12].

To meet the growing demand for food due to population increase, it becomes essential to cultivate crops in soil with high salt content or irrigate them using water with high salt concentrations. This is particularly important in regions where water resources are frequently scarce [13]. Soil salinization is a highly detrimental abiotic stress that affects numerous cultivated crops globally [14]. It impacts about 20% of the cultivated area globally, leading to reduced plant growth and thus decreasing crop production [15]. The expansion of the salt-affected regions primarily arises from a blend of natural and human-induced factors, including insufficient and inadequate precipitation, elevated temperatures, excessive evapotranspiration, and subpar water and quality irrigation management [16]. Soil salinity substantially negatively impacts crop productivity, especially in vegetable crops. This is because vegetable crops often exhibit a limited capacity to withstand the adverse effects of salt stress [17]. Karimzadeh et al. [18] discovered that the combined influence of drought and salinity had a detrimental impact on the morpho-physiological characteristics of tomato seedlings. Salinity and drought conditions trigger the production of reactive oxygen species in cellular compartments, including mitochondria, peroxisomes, and chloroplasts. In addition, reactive oxygen species play a significant role in causing suboptimal plant growth and reduced productivity due to the oxidation of lipids in cellular membranes and the degradation of nucleic acids and enzyme proteins [19].

Biochar could promote long-term production and improve fertilizer and water utilization efficiency [20]. International Biochar Initiative defined biochar as a finely textured organic substance with significant carbon. It is generated by the process known as pyrolysis, which includes thermal decomposition of feedstock at temperatures ranging from 300 to 600 °C in the presence of limited or no oxygen [21]. Biochar utilization in agricultural systems has garnered attention in recent years because of its potential advantages in enhancing crop productivity and environmental conditions [22]. According to Haddad et al. [23], using fertilizers and biochar are also primary methods for improving water use efficiency, soil fertility, and crop yields in water-limited regions. These methods help mitigate the detrimental effects of water stress. Furthermore, biochar improves soil physical characteristics, including water retention capacity, bulk density, porosity, and fertility [24]. Biochar enhances soil moisture retention, decreasing osmotic and oxidative stress, promoting plant growth and facilitating water absorption via plants [25]. Biochar utilization enhances soil water availability by modifying soil composition and augmenting its water retention capacity [26]. Biochar can enhance the health of sandy soil impacted by salt under arid environments, resulting in increased growth and yield of vegetation and improved water use efficiency in tomatoes [27]. Zahedifar et al. [28] reported that biochar addition positively influenced low-quality soil, enhancing the growth characteristics, biomass, and yield of crops under water and salt-induced stress. According to another research, when biochar was applied at a dosage of 4.8 t/ha, it caused a rise in the quantity of leaves, flowers, and the size of tomato fruits [29]. However, this increase was insufficient to compensate for the decrease in fruit production and the elevated sodium ion levels accumulated in the root system due to salt stress [29]. The main aim of utilizing biochar is contingent upon various elements, including soil composition, the quantity of biochar incorporated into soil and physical and chemical attributes of the biochars, which largely rely on pyrolysis parameters and the feedstock material used [30].

Many studies focused on investigating the impacts of either salinity or drought stress. However, only a limited number of studies examined the combined effects of both salinity and drought stress, and these studies revealed conflicting results, particularly about the utilization of various types of biochars.

Hence, the main aims of this research were to examine the impact of drought and salt-induced stress on the morphological and physiological characteristics, water use efficiency, and tomatoes yield. Additionally, the study aimed to determine if applying biochar derived from sesame residue might mitigate the adverse impacts of salt and water stresses.

## Materials and methods

### Research site and experimental design

The experiment was carried out from October 2022 to July 2023 in a greenhouse at Islamia University of Bahawalpur located in Bahawalpur, Punjab, Pakistan (29° 23’ 44.5956’’ N and 71° 41’ 0.0024’’ E). The weather pattern in District Bahawalpur is marked by scorching and arid summers, accompanied by prevailing dry and chilly conditions in winter. The maximum temperature increases to 48ºC, while the minimum temperature decreases to 7ºC. Summer often has numerous wind and dust storms. The region receives an average annual rainfall of 200 mm.

A three-factorial experiment was conducted with two water quality treatments. Sodium chloride (NaCl) was added to achieve salinity levels of 0.9 and 2.4 dS m^− 1^. Four deficit irrigation levels 40% ETc, 60 ETc, 80 ETc, and 100% of total evapotranspiration (ETc) were investigated. Additionally, biochar was applied at a rate of 3% (w/w) (equivalent to 2.20 kg m^− 2^) (BC3%), while untreated soil served as the control (BC0%). The control treatment involved complete irrigation (100% of full irrigation) without the addition of biochar or salinity.

Experiments were designed as a randomized complete block (split-split-plot design) with three replicates. Water quality was identified as the primary factor, with irrigation levels serving as sub-factors within it. Correspondingly, biochar addition located in sub-sub-plots, The overall experiment was consisted of 48 experimental units, distributed as follows; 2 water quality × 4 irrigation levels × 2 biochar × 3 replicates. The experimental unit consists of a line 6 m length and 1 m width, with emitters spaced 0.4 m (15 plants) and 1 m between the experimental units. The control was full irrigation (100% ETc) without salinity and biochar.

The research was conducted using the commercial tomato variety ‘NIAB tomato-21’ in a greenhouse environment. Tomato seeds were planted in foam pots filled with a mixture of vermiculite and peat moss (in a 1:1 ratio by volume) on October 20, 2022. In a controlled environment within a fiberglass greenhouse, seedlings are grown using standard procedures at a daytime temperature of 25 °C and an at-night temperature of 20 °C to protect them from cold weather. After four weeks the seedlings were moved to the control greenhouse, where they were standardized to consistent size with five leaves. The control greenhouse maintained a temperature of 26 °C during the day, 19 °C at night, and a relative humidity of 75%. The agricultural practices commonly advised for commercial tomato production in greenhouses were utilized, which encompassed soil sterilization, insect management, and fertilizer. The local farmers followed the required application rates of 238 kg potassium, 142 kg phosphorus, and 285 kg nitrogen per acre for fertilizers during the growing seasons [17]. The greenhouse was the designated location for installing the surface drip irrigation system. The irrigation levels were selected based on the daily evapotranspiration and crop coefficient (Kc) standards. These levels were set at 40%, 60%, 80%, and 100% of the crop’s water requirement (ETc). The ETc was determined using the following formula (Allen et al. [77]

1 $$ETc=Eo\times Kp\times Kc$$

The variables in the equation are Eo, which represents the evaporation from pan A in millimeters; Kp, which represents the pan coefficient; and Kc, which represents the crop coefficient.

### Soil and water analysis

Before the experiment, water and soil samples were collected from the greenhouse. A sample of sandy soil was air dried, passed through a 2 mm sieve, and a saturated soil paste extract was prepared. Analyzes of the water and soil samples, including the pH and EC, were performed using a pH (CG 817) and an EC (Test Kit Model 1500-20, Cole and Parmer) meter. Water-soluble sodium (Na^+^), magnesium (Mg^2+^), potassium (K^+^), calcium (Ca^2+^), and chloride (Cl^−^) were measured using an ion chromatography device (ICS-5000, Thermo Fisher Scientific, Waltham, MA, USA). Bicarbonate (HCO_3_^−^) and soluble carbonate (CO_3_^2−^) were measured using a titration method [31]. The soil and water undergo chemical analysis; the results are displayed in Table 1.

**Table 1:** Chemical and physical characteristics of water and soil

Parameters	Soil	Saline water	Fresh water
pH	7.16 ± 0.04	7.61 ± 0.04	7.03 ± 0.03
Electrical conductivity (dSm^− 1^)	2.57 ± 0.05	2.29 ± 0.06	0.88 ± 0.04
CEC (cmolckg^− 1^)	11.60 ± 0.30	-	-
Sodium (meql^− 1^)	3.91 ± 0.05	22.31 ± 0.13	5.02 ± 0.03
Potassium (meql^− 1^)	4.99 ± 0.06	0.31 ± 0.25	0.09 ± 0.01
Magnesium (meql^− 1^)	2.31 ± 0.05	2.18 ± 0.05	2.61 ± 0.05
Calcium (meql^− 1^)	11.0 ± 0.07	2.79 ± 0.05	3.21 ± 0.05
Soluble carbonate (meql^− 1^)	0	0	0
Bicarbonate (meql^− 1^)	19.1 ± 0.10	2.97 ± 0.05	2.28 ± 0.05
Chloride (meql^− 1^)	4.49 ± 0.05	23.06 ± 0.15	8.20 ± 0.03
Calcium carbonate equivalent %	56 ± 3.50	-	-
Organic matter %.	0.50 ± 0.08	-	-
Sand %	59 ± 1.05	-	-
Silt %	31 ± 0.25	-	-
Clay %	13 ± 0.10	-	-
Soil texture	Sandy loam	-	-
Available P (mg kg^− 1^)	14 ± 0.02	-	-
Available K (mg kg^− 1^)	253 ± 3.50	-	-

### Production of Biochar

The biochar utilized in this experiment was derived from sesame residue. Biochar production involved heating the material to pyrolysis at a temperature of 550 °C for 2 h. The physico-chemical characteristics of biochar have been examined after its manufacture and are presented in Table 2.

**Table 2:** The physicochemical characteristics of biochar

Parameters	Biochar properties
pH	9.01 ± 0.03
Surface area (m2g^− 1^)	241.72 ± 3.50
Electrical conductivity (dSm^− 1^)	4.03 ± 0.06
Organic matter %	29.41 ± 2.01
Nitrogen %	0.23 ± 0.01
Phosphorus %	0.19 ± 0.01
Potassium %	0.93 ± 0.01
Calcium %	0.59 ± 0.01
C: N ratio	249:1 ± 3.01
Moisture %	4.13 ± 0.01
Ash %	26.34 ± 0.12

### Measurement of growth-related parameters and physiological aspects

Plant growth-related attributes were assessed, such as the plant’s height, leaf area index, and diameter of stem, as well as the plant’s dry and fresh weight (containing both stems and leaves). Dry weight was measured using a digital balance assessment after the sample was desiccated at a temperature of 70 °C until a uniform dry weight was achieved, employing a convection oven. Leaf tissues were utilized to determine the leaf’s relative water content, which was defined as follows: the discs of leaves were collected to assess fresh weight, and then they were immersed in deionized water for a maximum of 4 h to get turgid weight. Dry mass was measured by placing the leaves in oven drying at around 85 °C till they attained a consistent weight. Leaf’s relative water content was determined by applying the methodology described by Smart and Bingham [32].

2 $$\text{L}\text{e}\text{a}\text{f}\,\text{R}\text{e}\text{l}\text{a}\text{t}\text{i}\text{v}\text{e}\,   \text{W}\text{a}\text{t}\text{e}\text{r}\,  \text{C}\text{c}\text{o}\text{n}\text{t}\text{e}\text{n}\text{t}=\frac{\text{f}\text{r}\text{e}\text{s}\text{h}\, \text{w}\text{e}\text{i}\text{g}\text{h}\text{t}-\text{d}\text{r}\text{y}\,  \text{w}\text{e}\text{i}\text{g}\text{h}\text{t}}{Turgid\,weight-dry\,weight}\times 100$$

### Measurement of LAI

The LAI values were measured throughout the growing period starting at 40 days after transplanting in 3 replications in each treatment at 10-day intervals and lasted until 4 times using the AccuPAR ceptometer LP-80, Decagon Devices Germany. The ceptometer is a battery-operated menu driven device, which is used to measure light interception in plant canopies to calculate LAI. Its main components are an integrated microprocessor-driven data logger and a probe with 80 sensors. Data were collected from menu screen by inserting the probe into canopy. For determining LAI in a destructive way, all leaves were removed separately from randomly selected 3 plants from each treatment at the last sampling date. The collected leaves (tomato, 10–12 per plant) were placed into a rectangular sketch of a white paper.

Three completely matured leaves from the uppermost layer of plants were chosen per each experimental part to determine the transpiration and photosynthetic rate and conductivity. The photosynthetic rate measurement was estimated in a closed system of infrared gas analyzer Li-Cor 6400 Portable Photosynthesis system. Before warming and calibrating the portable photosynthesis system. In the first step, the initial zeroing process for the built-in flow meter and the second step for the infra-red gas analyzer were observed. The measurements were used for optimal cuvette conditions such as 1000 Kumol photosynthetically active radiation (PAR), 400 µmol/ mol carbon dioxide, 30 °C leaf temperature, and 60% relative humidity with air flow rate of 500 cm³/min. The measurements of gas exchange were carried out between 9:00 to 11:00 a.m. The leaf surfaces were cleaned and dried before enclosed in the leaf cuvette. Data for photosynthesis rate and transpiration rate were simultaneously recorded. The spectrophotometric determination of chlorophyll a and b, carotenoids, and total chlorophyll is conducted using the method described by [33]. Leaf chlorophyll content was determined by using the Coombs method [33]. Leaves were gnawed using cock borer to get four sample areas of 1 cm² per gnawing. Samples were put into a vial, and 20 ml of 80% (v/v) acetone was poured into a vial and covered with aluminum foil. These samples were kept in a dark place for about three to seven days until extraction of all chlorophyll from leaves. Chlorophyll content was then determined using Spectrophotometer (Model UV 3101 PC) at wavelengths of 664 nm and 647 nm. The values for Chlorophyll a and b, carotenoids, and total chlorophyll were determined using the below Eq. 

3 $$\text{C}\text{h}\text{l}\text{o}\text{r}\text{o}\text{p}\text{h}\text{y}\text{l}\text{l} \text{a}=\left[\left(12.7\times \text{O}.\text{D} 663\right)-\left(2.69\times \text{O}.\text{D}645\right)\right]\times \frac{\text{V}}{1000}\times \text{W}$$

4 $$\text{C}\text{h}\text{l}\text{o}\text{r}\text{o}\text{p}\text{h}\text{y}\text{l}\text{l} \text{b}=[\left(22.9\times \text{O}.\text{D}645\right)-\left(4.68\times \text{O}.\text{D}663\right)]\times \text{V}/100$$

5 $$\text{T}\text{o}\text{t}\text{a}\text{l} \text{c}\text{h}\text{l}\text{o}\text{r}\text{o}\text{p}\text{h}\text{y}\text{l}\text{l}=\left[\right(20.2\times \text{O}.\text{D}645+\left(8.02\times \text{O}.\text{D}663\right)]\times \text{V}/1000\times \text{W}$$

6 $$Carotenoids=[O.D480+\left(0.114\times \text{O}.\text{D}663\right)]\times (0.638\times \text{O}.\text{D}645)$$

O.D. the extract’s optical density at the specified wavelength and V represents the extract’s volume, measured in milliliters (mL). W: mass of the leaves when they are freshly harvested (g) [34]. The amounts of proline in leaves were estimated using Clausen’s technique [35].

### Water use efficiency and total yield

The total yield and each fruit weight were determined with digital balances during the entire harvesting period, measured in kilograms per square meter. The water use efficiency (WUE) was determined by dividing total fresh fruit yield (TFFY in kilograms) by the cumulative quantity of the water provided (CIW, in cubic meters) to the tomato plants over the entire planting season, as stated by Lovelli et al. [36].

7 $$Water\,Use\,Efficiency\,(kg/m3)=TFFY/CIW$$

Yield decline (YR %) and saved water (%) were calculated with Eqs. (8), (9) from the study conducted by [37]. The enhancement in WUE was computed by applying Eq. (10), as per the study conducted by [38].

8 $$YR\%=\left[\frac{(yeild\,of\,control-yeild\,of\,treatment)}{yeild\,of\,control}\right]\times 100$$

9 $$Water\,saving \%=\left[\right(WCC-WCT) /WCC ]\times 100$$

WCC represents the use of water by control group, determined in m_3_/m^2^. WCT represents the water consumption of the treatment group, also determined in m^3^/m^2^.

10 $$Improved WUE\%=\left[\frac{(WUE of treatment-WUE of control)}{WUE of control}\right]\times 100$$

### Statistical evaluation

Data was statistically analysed by applying ANOVA with SAS software. The revised least significant difference (LSD) test was conducted at a confidence level of 0.05, as stated by Steel and Torrie [39].

## Results

### Morphological characteristics of tomato plants

High salinity levels and water stress adversely impact several plant growth-related parameters, such as plant height, stem diameter, leaf area, and dry and wet weight. Conversely, using biochar enhanced all plant growth components (Table 3). In this concern, tomato plants under salt stress had lower (*P* ≤ 0.05) the aforementioned parameters than fresh watered plants by 15.1%, 19.4%, 91.8%, 21.0%, and 12.8%, respectively. A similar decrease was seen when the deficiency irrigation levels were applied in comparison to fully irrigated plants. The irrigation stress leads to a notable reduction in most of the morphological traits, depending upon the period and level of the stress [3]. Our results revealed that, the most reduction was achieved at irrigation levels of 60% or 40% applied, lowered the previously indicated growth-related parameters by 22.7 or 29.6%, 23.7 or 40.2%, 17.5 or 28.8%, 26.5 or 37.0% and 16.0 or 28.9%, orderly, compared to full 100% irrigated plants. The plant’s vegetative growth properties were affected by the presence of saline water, which resulted in a nutritional imbalance. Furthermore, a high salt content caused poor plant growth, mainly due to ion toxicity and osmotic stress [8]. Conversely, biochar (BC_3_%) significantly improved tomato growth-related parameters in the current study’s region, increasing plant height by 6.8%, stem diameter by 7.6%, leaf area by 9.0%, dry weight by 6.3%, and wet weight by 5.3% in comparison to non-added biochar (BC_0_%).

The interaction among water stress, biochar, and salinity substantially impacted plant height, stem diameter, leaf area index, and fresh and dry weights, as shown in Table 4. The biochar addition positively affected the vegetative growth characteristics across all irrigation levels, mainly when fresh water was used for irrigation. It’s interesting to note that the highest growth improvements in plant height (7.4% and 7.3%), stem diameter (5.7% and 13.6%), leaf area index (4.7% and 7.8%), fresh weight (6.2% and 4.8%), and dry weight (8.8% and 6.5%), respectively, were observed under irrigation level at 100% ETc and received biocahr at (BC_3_%), compared to non-added (BC_0_%) biochar plants. This was observed when comparing fresh water (0.9 dS m^− 1^) and saline water (2.4 dS m^− 1^), orderly. The positive impacts of biochar on vegetative growth characteristics are ascribed to its ability to stimulate microbial activity within the root zone and improve the soil’s capacity to retain water [11]. Furthermore, the biochar exhibits a substantial concentration of minerals, including calcium, magnesium, and inorganic carbon, which provide beneficial impacts on the growth of plants [12]. The application of biochar resulted in an improvement in the water status of the soil and a reduction in ion concentration in the presence of salt stress, so creating a conducive environment for the growth of plants. Additionally, the incorporation of biochar led to enhanced vegetative growth as a result of mitigating oxidative and osmotic stressors [13]. Conversely, the biochar addition with saltwater led to reduced vegetative growth attributes, particularly when the *Solanum lycopersicum* crop was exposed to drought-induced stress at 40% ETc and 60% ETc (Table 4). As it would be proposed, exactly how much biochar is used determines how much improvement it may achieve. For this reason, the adverse reaction to saline water could be due to the low biochar dosage (BC3%) treatment (Thomas et al., 2013).

**Table 3:** The impacts of water salinity, irrigation and biochar, on tomato plant morphological characteristics

Treatments	Plant height (cm)	Stem diameter (mm)	Leaf area index	Plant fresh weight (kg)	Plant dry weight (g)
**Salinity (dS m** ^**− 1**^ **)**					
**0.9**	329.06 ± 3.86a	16.37 ± 0.88a	8.81 ± 0.04a	1.86 ± 0.05a	217.02 ± 6.33a
**2.4**	279.53 ± 0.79b	13.19 ± 0.88b	0.72 ± 0.03b	1.47 ± 0.09b	189.14 ± 11.87b
**Irrigation levels (ETc %)**					
**40**	254.17 ± 4.92a	11.01 ± 1.45a	0.57 ± 0.02a	1.19 ± 0.05a	169.21 ± 4.38a
**60**	279.14 ± 4.59b	14.06 ± 0.88b	0.66 ± 0.03b	1.39 ± 0.09b	200.11 ± 6.88b
**80**	329.97 ± 3.86c	15.36 ± 0.88c	0.72 ± 0.03c	1.68 ± 0.27c	219.47 ± 6.39c
**100**	361.01 ± 3.21d	18.42 ± 0.88d	0.80 ± 0.05d	1.89 ± 0.05d	238.14 ± 3.79d
**Biochar (%)**					
**BC0%**	299.31 ± 4.12b	14.08 ± 0.88b	0.71 ± 0.03b	1.49 ± 0.02b	204.36 ± 5.40b
**BC3%**	321.09 ± 3.92a	15.24 ± 0.88a	0.78 ± 0.03a	1.59 ± 0.06a	215.87 ± 6.88a

**Table 4:** The study examined the interactive impacts of salinity, biochar, and deficit irrigation on various morphological features of tomato plants

Salinity level (ds m^− 1^)	Irrigation level (%)	Biochar treatment	Plant height (cm)	Stem diameter (mm)	Leaf area index	Plant wet weight (kg)	Plant dry weight (g)
**0.9**	**40**	**BC0%**	259.41 ± 3.1i	11.41 ± 1.45hi	0.59 ± 0.02 h	1.41 ± 0.02 h	182.13 ± 0.75 h
		**BC3%**	289.15 ± 0.4 h	12.57 ± 0.88 h	0.68 ± 0.06 g	1.48 ± 0.09gh	200.12 ± 3.05 fg
	**60**	**BC0%**	309.63 ± 14.3 g	13.07 ± 0.88 fg	0.69 ± 0.06 fg	1.56 ± 0.06f	209.27 ± 1.50ef
		**BC3%**	328.45 ± 0.8f	15.99 ± 0.88d	0.77 ± 0.02 cd	1.71 ± 0.05e	216.41 ± 4.42e
	**80**	**BC0%**	350.01 ± 5.8de	14.98 ± 0.88de	0.70 ± 0.02ef	1.81 ± 0.05d	222.24 ± 4.42de
		**BC3%**	365.74 ± 10.0b	18.88 ± 0.05ab	0.80 ± 0.03bc	2.10 ± 0.04ab	266.61 ± 6.75a
	**100**	**BC0%**	362.50 ± 10.0bc	17.97 ± 0.88	0.82 ± 0.03b	1.98 ± 0.03b	231.67c
		**BC3%**	391.48 ± 24.8a	19.06 ± 0.05a	0.86 ± 0.03a	2.11 ± 0.04a	254.11ab
**2.4**	**40**	**BC0%**	241.22 ± 5.2j	10.49 ± 0.88j	0.54 ± 0.02i	1.04 ± 0.07k	159.34 ± 5.99i
		**BC3%**	218.27 ± 10.1k	9.41 ± 0.1k	0.48 ± 0.02j	0.98 ± 0.07I	152.54 ± 5.69j
	**60**	**BC0%**	254.67 ± 6.8i	11.24 ± 1.45i	0.63 ± 0.02 h	1.19 ± 0.06i	183.23 ± 0.92 h
		**BC3%**	237.23 ± 10.1j	10.78 ± 1.45ij	0.55 ± 0.02i	1.11 ± 0.04j	179.38 ± 2.39hi
	**80**	**BC0%**	303.74 ± 4.3gh	13.30 ± 0.62 g	0.66 ± 0.03 fg	1.51 ± 0.06 fg	196.93 ± 6.16 g
		**BC3%**	311.41 ± 2.5 g	12.21 ± 1.03 h	0.65 ± 0.03 g	1.57 ± 0.06f	210.34 ± 1.50ef
	**100**	**BC0%**	331.49 ± 0.8ef	14.54 ± 0.88ef	0.71 ± 0.02de	1.80 ± 0.03de	229.31 ± 3.08 cd
		**BC3%**	357.41 ± 10.0 cd	16.83 ± 2.63c	0.77 ± 0.02c	1.89 ± 0.03c	245.28 ± 6.75bc

### Physiological parameters

**Fig. 1:**
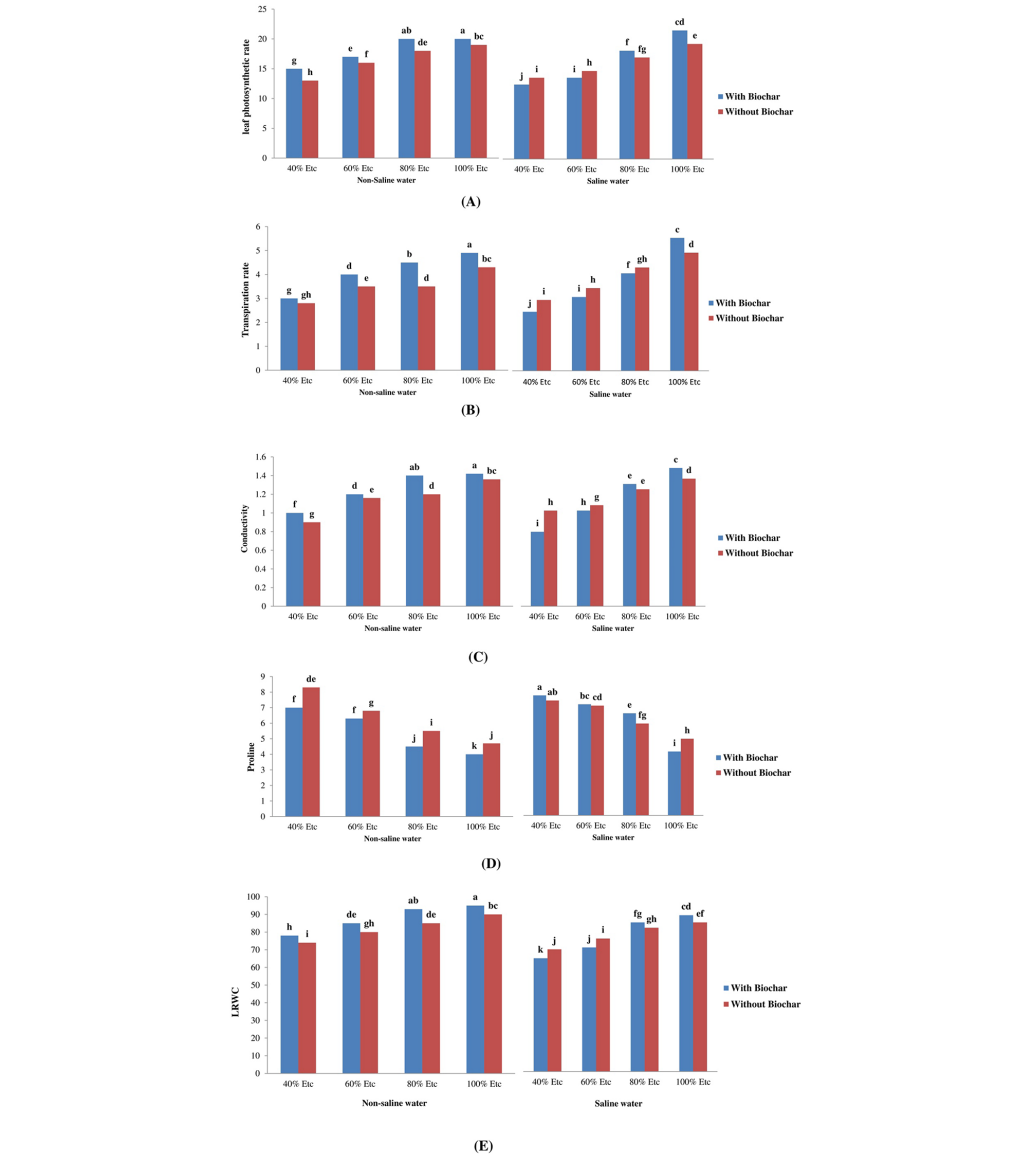
Combined impacts of biochar, deficit irrigation, and salinity on various parameters of tomato leaf rate of photosynthesis (µmolCO_2_m^− 2^S^− 1^) (**A**), rate of transpiration (mmolH_2_OCO_2_m^− 2^S^− 1^) (**B**), conductivity (mmolH_2_OCO_2_ m^− 2^S^− 1^) (**C**), proline levels (mg/g^− 1^ FW) (**D**), and leaf relative water content (%) (**E**)

The gas exchange of leaf attributes (photosynthesis, rate of transpiration conductivity, and leaf relative water content) were significantly decreased by drought, salt, and stresses, especially when the irrigation was 60 and 40% compared to 80 and 100%. When compared to fresh water, there was a significant drop in the aforementioned leaf gas exchange properties by 15.8%, 21.4%, 4.3%, and 10.7% when the tomato plant was irrigated with low quality (2.4 dS m^− 1^) water. Accordingly, the tomato plant was exposed to 60% ETc or 40% ETc, respectively, resulting in a significant decrease of 23.1% or 6.5%, 26.1% or 39.6%, 28.4% or 45.4%, and 13.3% or 21.2% of the aforementioned leaf gas exchange properties as compared to full irrigated (100% ETc) plants. The proline concentration in the leaves increased with salt and water deficit irrigation (2.4 dS m^− 1^ and at 60% or 40 ETc, orderly), comparatively to the control, Table (5). Under such circumstances, proline increased by 33.6% due to saline (2.4 dS m^− 1^) water, and in response to water deficit irrigation (i.e. 60% or 40 ETc, respectively), by 63.7% or 79.8%, relative to control plants. On the other hand, the incorporation of biochar at a concentration of 3% led to the most favorable leaf gas exchange characteristics, LRWC, augmenting by 5.2%, 4.8%, 9.9%, 2.5%, respectively, and the least amount of proline (dropped by 3.6%), in the tomato leaves as compared to plants that were not treated (Table 5). The incorporation of 3% biochar into freshwater resulted in the most significant improvements in leaf gas exchange characteristics across all water deficit treatments and when 100% ETc was added, as compared to the untreated plants without biochar. Conversely, the combination of salinity and deficit with 40% ETc and 60% ETc had a negative impact on all leaf gas exchange traits (Fig. 1A–C). The findings depicted in Fig. 1D demonstrate that the leaves of tomatoes cultivated under biochar with saline water exhibited the highest proline level at the maximum water deficit of 40% ETc. Conversely, the leaves watered with fresh water at 100% ETc displayed the lowest proline content. The irrigation levels with biochar and freshwater yielded the greatest LRWC values, surpassing those of the untreated plants (without biochar). In contrast, the lowest values for LRWC were found with biochar and irrigation with saline water under the highest water deficits of 40% and 60% ETc (Fig. 1E).

**Table 5:** The impact of irrigation levels, biochar, and salinity levels on the physiological characteristics of tomato leaves, such as leaf gas exchange attributes, proline concentration, and leaf relative water content (LRWC).

Applications	Photosynthetic level (µmol CO_2_ m^− 2^S^− 1^)	Transpiration level (mmol H_2_O CO_2_ m^− 2^S^− 1^)	Conductivity (mmol H_2_O CO_2_ m^− 2^S^− 1^)	LRWC %		Proline content (mg/g^− 1^ FW)
**Salt level dS m** ^**− 1**^						
0.9	18.16 ± 0.19^a^	3.98 ± 0.08^a^	1.19 ± 0.05^a^	85.37 ± 3.29^a^	5.89 ± 0.10^b^	
2.4	15.29 ± 0.11^b^	3.13 ± 0.08^b^	1.02 ± 0.05^b^	76.23 ± 0.46^b^	7.87 ± 0.02^a^	
**Irrigation levels %**						
40	12.49 ± 0.91^d^	2.59 ± 0.17^d^	0.77 ± 0.03^d^	71.15 ± 1.44^d^		9.01 ± 0.12^d^
60	14.61 ± 0.92^c^	3.17 ± 0.05^c^	1.01 ± 0.05^c^	78.24 ± 0.57^c^		8.34 ± 0.10^c^
80	17.31 ± 0.57^b^	3.48 ± 0.05^b^	1.18 ± 0.05^b^	85.56 ± 3.29^b^		6.13 ± 0.16^b^
100	19.01 ± 0.40^a^	4.29 ± 0.05^a^	1.41 ± 0.09^a^	90.28 ± 3.05^d^		5.01 ± 0.26^a^
**Biochar**						
BC_0%_	16.33 ± 0.11^b^	4.42 ± 0.07^b^	1.09 ± 0.06^b^	80.13 ± 2.19^b^		8.03 ± 0.24^a^
BC_3%_	17.18 ± 0.57^a^	4.63 ± 0.05^a^	1.21 ± 0.06^a^	82.19 ± 3.29^a^		7.75 ± 0.01^b^

### Photosynthetic pigments

The photosynthetic pigments feature, including the leaf index (SPAD), total chlorophyll, chlorophyll a, chlorophyll b, and carotenoids decreased in tomato exposed to salinity and drought stress, as shown in Table 6. Drought and salt stress have been shown to reduce the content of photosynthetic pigments. It’s found that, irrigating tomato with low quality of water (2.4 dS m^− 1^), reduced the aforementioned photosynthetic attributes by 18.2%, 11.4%, 11.1%, 18.3%, and 14.1%, respectively, in comparison to fresh water (0.9 dS m^− 1^) control. In respect deficit treatments, the most significant reduction was attained when exposed tomato plants to the most severe drought of 60% ETc or 40% ETc levels, resulting in decreases by 25.7 or 37.0%, 21.7 or 27.9%, 14.8 or 24.7%, 20.3 or 25.4%, and 14.7 or 26.5% for the correspondingly photosynthetic characteristics, compared to those plant received 100% ETc of water capacity.

Conversely, the incorporation of biochar significantly increased the leaf green index by 7.1%, total chlorophyll by 3.0%, chlorophyll a and b (by 7.9% and 5.8%, orderly), and carotenoids by 4.7%, in comparison to the plants that were not treated (BC_0_%) (Table 6). Plants that were treated with biochar and irrigated with fresh water at 100% of ETc exhibited the highest values for leaf pigment traits. In contrast, plants irrigated with saline water, particularly under the maximum water deficit of 40% ETc, had the lowest values (Table 7). The experimental findings demonstrate that the utilization of biochar leads to an enhancement in the rate of photosynthesis, hence suggesting a boost in the concentration of chlorophyll.

**Table 6:** Impact of salinity, irrigation and biochar, on the leaf index, total chlorophyll, chlorophyll a and b, carotenoids in tomato plants

Treatments	Index of green leaf (SPAD)	Total chlorophyll (mg/g^− 1^)	Chlorophyll a (mg/g^− 1^)	Chlorophyll b (mg/g^− 1^)	Carotenoids (mg/g^− 1^)
**Salinity (dS m**^**− 1**^ )					
**0.9**	49.22 ± 3.52a	3.59 ± 0.06a	2.44 ± 0.17a	1.09 ± 0.14a	4.88 ± 0.05a
**2.4**	40.27 ± 2.37b	3.18 ± 0.08b	2.17 ± 0.00b	0.89 ± 0.063b	4.19 ± 0.05b
**Irrigation levels %**					
**40**	34.01 ± 0.95d	2.89 ± 0.18d	2.13 ± 0.05d	0.88 ± 0.034d	3.80 ± 0.05d
**60**	40.12 ± 2.37c	3.14 ± 0.13c	2.41 ± 0.04c	0.94 ± 0.034c	4.41 ± 0.06c
**80**	47.29 ± 0.83b	3.72 ± 0.13b	2.62 ± 0.005b	1.11 ± 0.047b	4.91 ± 0.05b
**100**	54.02 ± 1.55a	4.01 ± 0.11a	2.83 ± 0.03a	1.18 ± 0.047a	5.17 ± 0.05a
**Biochar**					
**BC0%**	43.24 ± 1.66b	3.42 ± 0.13b	2.42 ± 0.04b	1 ± 0.032b	4.51 ± 0.07b
**BC3%**	46.29 ± 0.89a	3.69 ± 0.12a	2.56 ± 0.01a	1.03 ± 0.032a	4.72 ± 0.07a

7.1%, 7.9%, 5.8%, 3.0%, and 4.7%

**Table 7:** Effects of the interaction between salinity, biochar, and deficit irrigation, on various plant parameters, including leaf index, total chlorophyll, chlorophyll a and b, and carotenoids in tomato plants

Salinity (dS m-1)	Level of irrigations (%)	Biochar (%)	Index of green leaf (SPAD)	Total chlorophyll (mg/g-1)	Chlorophyll a (mg/g-1)	Chlorophyll b (mg/g-1)	Carotenoids (mg/g-1)
**0.9**	**40**	**BC0%**	36.01 ± 2.88i	3.04 ± 0.01 g	2.09 ± 0.00i	0.83 ± 0.02f	4.01 ± 0.05i
		**BC3%**	39.27 ± 0.23 h	3.41 ± 0.04e	2.29 ± 0.05gh	1.03 ± 0.05de	4.36 ± 0.07 h
	**60**	**BC0%**	42.99 ± 0.19f	3.31 ± 0.07ef	2.26 ± 0.05 h	1 ± 0.05ef	4.71 ± 0.05 fg
		**BC3%**	45.39 ± 1.66e	3.81 ± 0.05bc	2.59 ± 0.01de	1.09 ± 0.02c	5.02 ± 0.04de
	**80**	**BC0%**	49.61 ± 0.57d	3.71 ± 0.02 cd	2.58 ± 0.01e	1.05 ± 0.02de	4.69 ± 0.05 fg
		**BC3%**	57.97 ± 2.36b	4.06 ± 0.06a	2.79 ± 0.005ab	1.31 ± 0.09ab	5.49 ± 0.05b
	**100**	**BC0%**	57.69 ± 2.36b	3.91 ± 0.04b	2.67 ± 0.01bc	1.10 ± 0.05 cd	5.29 ± 0.05c
		**BC3%**	61.02 ± 0.98a	4.21 ± 0.06a	2.91 ± 0.03a	1.30 ± 0.02a	5.81 ± 0.04a
**2.4**	**40**	**BC0%**	33.78 ± 0.13j	2.80 ± 0.05 h	1.89 ± 0.06k	0.59 ± 0.02i	3.59 ± 0.05j
		**BC3%**	29.11 ± 0.96k	2.49 ± 0.04i	1.86 ± 0.06k	0.90 ± 0.07 g	3.21 ± 0.05k
	**60**	**BC0%**	37.077i	3.09 ± 0.01 fg	2.23 ± 0.05 h	0.92 ± 0.07 g	4.09 ± 0.05hi
		**BC3%**	33.49 ± 0.24j	2.69 ± 0.01 h	2.03 ± 0.00j	0.66 ± 0.06 h	3.65 ± 0.05j
	**80**	**BC0%**	41.05 ± 1.66 g	3.29 ± 0.06e	2.27 ± 0.05 g	1 ± 0.05f	4.49 ± 0.06 g
		**BC3%**	44.61 ± 2.37e	3.60 ± 0.06d	2.49 ± 0.04f	1.02 ± 0.05d	4.59 ± 0.05 fg
	**100**	**BC0%**	47.2 ± 0.89de	3.69 ± 0.05 cd	2.63 ± 0.05de	1.07 ± 0.05de	4.79 ± 0.07ef
		**BC3%**	50.34 ± 1.92 cd	3.89 ± 0.05b	2.68 ± 0.05 cd	1.11 ± 0.11b	5.10 ± 0.05d

### Water Use Efficiency and Fruit Yield

The biochar addition, water quality (fresh and saline water), and irrigation deficiency all impact the total yield and water use efficiency (WUE) of tomatoes. These effects are summarized in Table 8. The findings revealed that incorporating biochar led to a significant increase in overall crop production and water use efficiency. By incorporating 3% biochar with fresh water, the tomato plants’ yield was enhanced by 4.6%, 16.7%, 8.6%, and 2.9% for 100%, 80%, 60%, and 40% ETc irrigation treatments, respectively, in comparison to the untreated plants (BC_0%_).

**Fig. 2:**
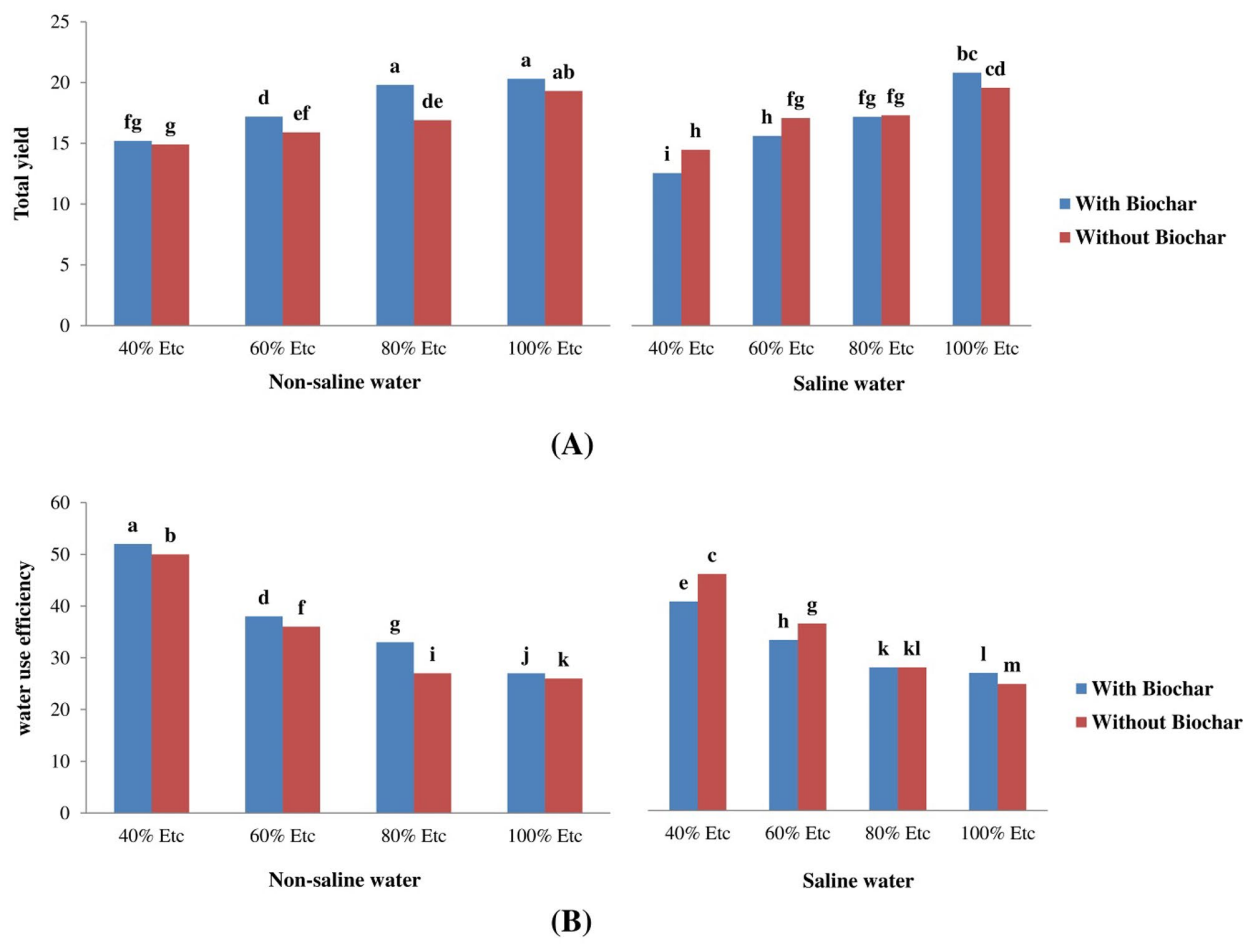
Combined impacts of biochar, water deficit, and salinity on the total fruit yield (kg m^− 2^) (**A**) and water usage efficiency (kg m^− 3^) (**B**) of *Solanum lycopersicum*

The water use efficiency (WUE) of tomato plants was observed to rise by 98% when they were exposed to biochar treatment and freshwater irrigation under a deficit irrigation of 40% ETc, as compared to full irrigation (Fig. 2). In comparison, the incorporation of biochar resulted in a decrease of 42% in tomato production when underwent saline water irrigation under the most extreme stress conditions (40% ETc), as depicted in Fig. 2.

**Table 8:** The impact of biochar, deficit irrigation, and salinity on yield reduction, water conservation, overall fruit yield, and water use efficiency in tomato plants

Applications	Applied total water (m^− 3^/m^− 2^)	Saved water (%)	Total yield (kg/m^− 2^ )	Yield decrease (%)	Water use efficiency (kg/m^− 3^)	Enhancement in water use efficiency (%)
Salt levels (dS m^− 1^)						
0.9	-	-	16.99 ± 1.29a	0.0	37.01 ± 1.45a	0.0
2.4	-	-	13.99 ± 0.02b	13.78 ± 0.02	31.14 ± 20.01b	-14.41
Irrigation levels						
40	0.297 ± 0.01	59.93 ± 1.60	13.49 ± 0.02d	29.01 ± 0.75i	45.29 ± 0.16a	78.99 ± 2.08a
60	0.423 ± 0.01	40.01 ± 1.19	15.51 ± 0.40c	17.46 ± 0.10i	35.02 ± 0.15b	37.06 ± 1.45a
80	0.589 ± 0.01	20.03 ± 0.70	16.69 ± 1.29b	12.06 ± 0.90	28.13 ± 0.85c	11.01 ± 0.30
100	0.7400.01	0.0	19.01 ± 0.8a	0.0	26.01 ± 1.50d	0.0
Biochar						
BC0%	-	-	16.03 ± 1.29b	0.0	34.15 ± 0.90b	0.0
BC3%	-	-	17.61 ± 0.35a	-2.67	34.91 ± 0.90a	1.23 ± 0.10

### Water Use Efficiency (WUE) improvement and Irrigation Water savings

The findings presented in Table 8 demonstrate that the use of salt water resulted in a 15% decrease in yield and a 16% decrease in WUE. The findings displayed in Table 8 indicate that a 40% ETc irrigation deficit resulted in a 28% decrease in tomato yield, but a 79% improvement in water use efficiency (WUE) compared to the control group with 100% ETc. The application of biochar at the stated rate (BC_3%_) resulted in a 2.9% increase in the yield and a 1.17% increase in the water use efficiency (WUE) of tomato plants. The observed enhancement in crop yield and water use efficiency (WUE) can be ascribed to the soil-based biochar behavior, which facilitates the growth of roots within the soil.

## Discussion

Abiotic stressors such as salinity can greatly affect plant growth, morphological characteristics, and biochemical and physiological features. If stress arises during the sensitive phases of plant life, it might reduce crop yield. Stress caused by salinity is a significant issue, especially in developing nations where people rely heavily on agriculture. Accumulated salts in the soil solution can create osmotic pressure, limiting water availability to plants [11]. Moreover, excessive accumulation of chloride and sodium can lead to an imbalance of ionic levels and toxicity to ions, which can hinder the uptake of other mineral nutrients through plants. Moreover, it can stimulate the production of abscisic acid and inhibit growth promoters [7]. The most common damages caused by salinity include imbalances in the ionic and water levels of the plant, reduced photosynthesis, and stomatal closure [2]. The imposition of irrigation stress resulted in a substantial reduction in the majority of morphological traits, contingent upon the intensity and duration of the stress [40]. The presence of saline water negatively affected the vegetative growth characteristics of plants, mainly caused by an imbalance in nutrition [41]. In addition, an elevated salt concentration resulted in insufficient plant growth, caused primarily by ion toxicity and osmotic stress [42]. The biochar addition led to an augmentation of nutrient availability, potentially improving plant morphology [43]. In addition, applying biochar in the soil increased water availability, hence mitigating the effects of osmotic stress [44]. Biochar supplementation mainly improves soil water retention and water holding capacity in soils, whereas it enhances water infiltration and drainage (saturated hydraulic conductivity) in fine-textured soils. Biochar application in soils considerably improves root systems and excellent roots, which enhances plants’ ability to bind soil particles. Biochar is crucial in soil and water conservation in dry and semi-dry areas [45]. The beneficial impacts of rice-derived biochar addition on the vegetative growth characteristics are ascribed to the expansion of the microbe’s growth in root areas and the improved capacity of soil for holding water [45]. Furthermore, biochar is rich in minerals, including inorganic carbon, magnesium, and calcium, which positively affect plant development [46]. Biochar incorporation improved the moisture level of the soil and reduced the concentration of ions in the presence of salinity stress, creating optimal conditions for the growth of plants [47]. In addition, the biochar addition enhanced plant growth by reducing osmotic and oxidative stressors [48]. Drought and salinization induce ionic and osmotic stress, thereby eliciting cellular stress responses and fostering the generation of reactive oxygen species (ROS) disrupting regular cellular activities. Abscisic acid (ABA) levels regulate the generation of reactive oxygen species (ROS), serving as a crucial chemical signal for plants to detect environmental stress and control crop development [43].

Several studies have shown the adverse impacts of drought and salinity on photosynthetic characteristics, leaf relative water content (LRWC), and growth of plants [49]. Increased salt content was observed to reduce gas exchange in *Solanum lycopersicum* seedling leaves, as observed by Abbas et al. [50]. Our results correlate with Rodrigues et al. [51], who found that the proline level was significantly enhanced by deficit irrigation and that a surge in proline percentage was linked to both salinity and drought [47]. On the other hand, when compared to plants that were not treated, *Solanum lycopersicum* leaves with 3% biochar added showed the most significant levels of leaf-relative water contents and the lowest levels of proline (Table 5). The increase in both gas exchange and LRWC, and the decrease proline content was due to the increasing water availability in the soil and salt leaching from the root zone. This reduces osmotic stress and enhances water uptake by the plant [52]. When 3% biochar was added to freshwater, the leaf gas exchange attributes showed the highest values in all drought treatments than control. Conversely, when drought and salinity were combined with 40% and 60% irrigation, all the leaf gas exchange characteristics were negatively impacted (Fig. 1A-C).

The findings shown in Fig. 1-D demonstrate that leaves of *Solanum lycopersicum* planted under biochar with salted water at maximum water stress (deficit) of 40% irrigation had the highest level of proline. In comparison, leaves watered with fresh water at 100% irrigation had the lowest level of proline. Compared to the control, the highest leaf-relative water content levels were recorded for all irrigation treatments using biochar-derived from sesame and fresh-water. Conversely, under the highest water shortages of 40% irrigation and 60% irrigation, saline irrigation and biochar produced the lowest values for leaf-relative water content (Fig. 1E). Massimi et al. [53] found that increasing the salt concentration decreased the transpiration rate by 70.55%, the stomatal conductance by 7.13%, and the photosynthetic rate by 72.34% in the leaves of tomato seedlings. According to the findings presented by Yang et al. [54], the addition of biochar significantly increased the photosynthetic rate (Ph), the relative water content (RWC), and recorded the lowest proline content in tomato plants exposed to a water deficit. Similarly, Rodrigues et al. [51] observed that adding biochar to stressed and unstressed tomato plants significantly improved the photosynthetic and transpiration rates. Additionally, the use of biochar improved the leaf gas exchange and LWRC under salinity and drought stress conditions, indicating that biochar helped the plants retain firm leaves under abiotic stresses [17]. In this study, the decreased chlorophyll could be due to damage to the thylakoid membranes, as a result of the destructive effect of reactive oxygen species (ROS) on chloroplasts [55]. The generation of reactive oxygen species (ROS) was significantly increased due to salinity and water deficiencies [50]. Another explanation for the observed reduction in chlorophyll concentration may be attributed to the detrimental effects of osmotic stress on the chloroplast layers, which leads to an increase in membrane permeability [56, 74]. For instance, previous studies have demonstrated that salt stress and drought can lead to a decrease in the concentration of photosynthetic pigments in tomato leaves [57, 58]. However, the incorporation of biochar led to a notable augmentation in the leaf green index, chlorophyll a, chlorophyll b, total chlorophyll, and carotenoids as compared to the plants that were not treated (BC_0%_) (Table 6). The findings were consistent with the results reported in references [59, 60]. Specifically, they observed that the biochar application increased chlorophyll levels when *Solanum lycopersicum* was exposed to drought and salt-induced conditions. Biochar’s effect on chlorophyll and carotenoid levels under salt stress is associated with increased antioxidant activity and the development of antioxidant capacity. Biochar supplementation stimulates the uptake of magnesium, an essential component in chlorophyll production [29]. Nazarideljou et al. [65] also discovered that the utilization of a 5% amendment (biochar) enhanced the productivity and growth attributes of *Solanum lycopersicum* cultivated in a salt-induced environment. According to the findings of our study, the utilization of biochar enhances the rate of photosynthesis, which is an indicator of elevated chlorophyll levels. Biochar enhances photosynthetic pigments by influencing nutrient intake and availability (potassium, phosphorus, magnesium, calcium, and sulfur) and enhancing soil’s physiochemical and biological characteristics [62, 63]. Biochar addition significantly enhanced the antioxidant activities, protecting the photosynthetic apparatus and pigments of plants from oxidative damage caused by salt stress [61].

The growth and yield of plants are negatively affected by salt and water stress, as evidenced by the findings of [64, 66]. Previous research has revealed that biochar incorporation can enhance plant growth, boost crop yields, and improve water use efficiency [67]. In a study carried out by Wang et al. [68], it was discovered that the incorporation of 50 tons ha^− 1^ of biochar resulted in a 55% increase in *Solanum lycopersicum* yield and a 45% improvement in water use efficiency than the control. Biochar treatment prevents membrane damage by reducing Na^+^ level and boosting K^+^, therefore enhancing leaf hydration status during salt stress. Biochar enhances leaf water status by elevating potassium (K) concentration, which is a crucial osmoprotectant in plant tissues. Applying biochar to plants enhances their leaf Relative Water Content, leading to an improved Water Use Efficiency by the plants [69].

When 3% biochar was added to freshwater, *Solanum lycopersicum* yield increased by 4.6%, 16.7%, 8.6%, and 2.9% for irrigation levels of 100, 80, 60, and 40%, respectively, then control. The water use efficiency (WUE) of *Solanum lycopersicum* plants increased by 97% when they were exposed to a deficit irrigation of 40% and supplemented with biochar. At the same time, they were supplied with fresh water. This is in comparison to *Solanum lycopersicum* plants that underwent full irrigation (Fig. 2). The increase in yield and WUE with the biochar might be explained by its ability to retain water, improve porosity, and provide nutrients to the plant under water stress conditions. The increase in WUE with deficit irrigation could be attributed to reductions in the transpiration rate (TR) and stomatal closure in response to salt and water stress [69].Conversely, the biochar addition resulted in a 42% decrease in *Solanum lycopersicum* yield when watered with saltwater under extreme stress-induced conditions (40%), as shown in Fig. 2, then control. It should be concluded that the negative effects from the biochar addition on the tomato yield in this study were most likely related to physiological drought resulting from the interaction between the biochar, saline water, and water deficit, and the high pH of biochar. As a result, the root absorption of water was more incomprehensible, leading to a decrease in the yield [70]. A high pH can affect the nutrient release into the soil, resulting in a decrease in the yield [71, 75, 76]. According to Bahadur et al. [72] the addition of biochar to the soil improved some vegetative growth attributes, but did not mitigate the negative effects of salt stress on tomato fruit yield. Table 8 shows that the use of saline water resulted in a 14% decrease in *Solanum lycopersicum* and a 15% decrease in water use efficiency. The data displayed in Table 8 indicates that exposing the *Solanum lycopersicum* plants to an irrigation deficit of 40% resulted in a 28% decrease in yield while simultaneously enhancing the water use efficiency by 79% than control. By incorporating biochar at a rate of 3%, the *Solanum lycopersicum* plants exhibited a 2.9% increase in yield and a 1.17% increase in water use efficiency. This increase in the yield and WUE can be attributed to biochar behavior in the soil, promoting root growth in the soil. Similar results were reported by Vajjiravel et al. [73] in the study on pepper plants grown in greenhouse, which indicated that the addition of biochar improved the WUE and irrigation water savings.

## Conclusion

Salinity and drought led to decreases in tomato’s growth-related characteristics, physiological parameters and productivity, due to damage from these stresses. In this sense, plant water status, photosynthetic efficiency, and corresponding chlorophyll pigment were all impacted by this outcome. Consequently, significant changes in plant development and growth by disrupting molecular, physiological, and biochemical processes are realised. Our findings indicating that, to effectively cultivate *Solanum lycopersicum* cultivars in semi-arid and arid areas with sandy soils and low agricultural productivity, it is necessary to add various amendments that help combat the detrimental impacts of drought and salinity. In this concern, the damage from these stresses can be ameliorated by the incorporation of 3% biochar, through positively improve the physio-morphological and functional traits and the water use efficiency of *Solanum lycopersicum* cultivated in a greenhouse environment. Biochar addition to sandy soil is a suggested technique to enhance the growth and production of tomatoes under drought and salinity conditions without any interaction between these two factors. Moreover, applying biochar enhances membrane stability, nutrient absorption, and nutrient balance, improving plant performance in salinity and drought stress conditions. Biochar limited the entry of harmful Na^+^ and enhanced the entry of K^+^ in response to salinity stress, which helps regulate stomata motions and enhance leaf gas exchange properties. In order to successfully adapt to the changing global climate, our results may help to develop strategies of the future applications to satisfying growth and yield with higher WUE of tomato under conditions impacted by salt and inadequate irrigation, particularly in arid and semiarid areas in Pakistan.

## Data Availability

The datasets analysed during this study are included in this manuscript.

## Declarations

### Statement on guidelines

All experimental studies and experimental materials involved in this research are in full compliance with relevant institutional, national and international guidelines and legislation.
